# Association of time spent in outdoor light and genetic risk with the incidence of depression

**DOI:** 10.1038/s41398-023-02338-0

**Published:** 2023-02-03

**Authors:** Jing Lin, Hongxi Yang, Yuan Zhang, Zhi Cao, Dun Li, Li Sun, Xinyu Zhang, Yaogang Wang

**Affiliations:** 1 School of Public Health, Tianjin Medical University, Tianjin, China; 2 Department of Bioinformatics, School of Basic Medical Sciences, Tianjin Medical University, Tianjin, China; 3 Raymond G. Perelman Center for Cellular and Molecular Therapeutics, Children’s Hospital of Philadelphia, Philadelphia, PA USA; 4 School of Public Health, Zhejiang University School of Medicine, Hangzhou, China; 5 School of Integrative Medicine, Tianjin University of Traditional Chinese Medicine, Tianjin, China; 6 School of Nursing, Tianjin Medical University, Tianjin, China; 7 School of Integrative Medicine, Public Health Science and Engineering College, Tianjin University of Traditional Chinese Medicine, Tianjin, China

**Keywords:** Depression, Human behaviour, Genetics

## Abstract

Depression is the consequence of both environment and genes working together. Genetic factors increase depression risk, but it is unclear whether this association can be offset by time spent in outdoor light. The study was undertaken to investigate the optimal time spent in outdoor light for lowering the risk of depression and the joint association of time spent in outdoor light and depression genetic risk. In UK Biobank, 380,976 depression-free individuals were included in this study. Polygenic risk score (PRS) was categorized into three groups in terms of tertiles. Time spent in outdoor light on a typical day in summer or winter originated from the questionnaire survey. Depression was defined as hospital admission. The potential dose-response relationship between time spent in outdoor light and depression risk was shown by a restricted cubic spline. Data were analyzed using Cox regressions and Laplace regression. After the median follow-up of 12.6 years, 13,636 individuals suffered from depression in the end. A nonlinear (J-shaped relationship) trend was observed between time spent in outdoor light and depression risk. On average, 1.5 h/day of outdoor light was related to the minimum risk of depression. Individuals below and above this optimal time both had elevated depression risk (below, HR = 1.09, 95% CI: 1.02–1.16; above, HR = 1.13, 95% CI: 1.07–1.20), and the time to incident depression were both shortened by 0.46 years (50th percentile differences [PD] = −0.46, 95% CI: −0.78, −0.14) and 0.63 years (50th PD = −0.63, 95% CI: −0.90, −0.35) years, respectively. In a comparison of individuals with the lowest tertile of PRS and average 1.5 h/day outdoor light, the HRs and 95% CIs of depression were 1.36 (1.21–1.53) and 1.43 (1.29–1.58) in those with the highest tertile of PRS and below/above this reference value, respectively. Significant multiplicative interactions were observed between intermediate genetic risks and longer time spent in outdoor light. We found that an average of 1.5 h/day spent in outdoor light was associated with a lower depression risk whatever the degree of depression genetic predisposition. Moderate time spent in outdoor light may contribute to a decreased depression risk even among people with a higher genetic risk of depression.

## Introduction

Depression is a common psychiatric health problem that profoundly influences an individual’s mental and physical function and may reduce the quality of life [1]. In 2019, depression ranked among the top 25 burden-causing diseases worldwide [2]. The World Health Organization predicts that the burden of depression will rank first by 2030 [3]. In recent years, the number of patients with depression has risen by 27.6% around the world, with an increase of 29.4% in major depression disorders seen in Central Europe [2]. Depression can lead to or contribute to many other health conditions, such as diabetes and cardiovascular disease. Depression may be also associated with an increased risk of mortality [4, 5]. Although the increasing prevalence of depression has been studied extensively, studies on preventive methods are scarce. Exploring the high-risk factors associated with depression may contribute to the prevention and early intervention.

Vitamin D may be a crucial factor in the incidence of psychiatric disorders like depression [6]. Although vitamin D can be taken in through the diet, few countries routinely fortify food with vitamin D [7]. Thus, outdoor light is commonly recommended, partially because outdoor light is freely available to many people. However, little research is available on the effects of time spent in outdoor light on the risk of depression, and the dose-response association between time spent in outdoor light and depression risk remains uncertain. Several previous studies have suggested a negative association between outdoor light and depression, indicating a short time spent in outdoor light could be related to an elevated risk of depression [8, 9]. A previous cross-sectional study from Korea reported a bimodal effect of outdoor light on depression [10]. Sufficient outdoor light decreases cortisol concentrations, which accelerates the synthesis of vitamin D [11, 12]. So far, however, it is not clear how long individuals should spend in outdoor light to experience a reduced risk of depression.

Depression is caused by various combinations of genetic and environmental risk factors. Previous studies have explored the genetic factors affecting depression based on the theory of polygenic inheritance [13, 14]. One analysis showed that the offspring of parents with a history of depression have a three- to four-fold greater risk of depression themselves than the offspring of healthy parents [15]. Genome-wide association studies (GWASs) have reported the risk genes for depression, including *B3GALTL*, *FADS1*, *TCTEX1D1*, *XPNPEP3*, *ZMAT2*, *ZNF501*, and *ZNF502* [16]. Polygenic risk score (PRS) was determined using these risk loci to indicate overall genetic susceptibility for depression [17]. So far, the evidence regarding whether time spent in outdoor light can affect the association between genetic predisposition and risk of depression risk has sparse.

Therefore, the major purpose of this study was to examine the association between time spent in outdoor light and depression risk and to investigate whether optimal time spent in outdoor light may compensate for depression-related genetic predisposition using data from the large-sample cohort of over half a million individuals.

## Materials and methods

### Study design and participants

Participants in UK Biobank were recruited began between 2006 and 2010, across England, Scotland, and Wales, and have been following up to September 30, 2021. Our study was restricted to White British people with the genetic information available and without a history of depression that was inpatient records and/or self-reported. Among 502,507 participants, we excluded a total of 121,531 participants. Our final analytic sample included 380,976 participants aged 38–73 years at baseline (Supplementary Fig. S1).

### Measurement of outdoor light

The data on outdoor light was collected at baseline assessment (2006–2010) by a questionnaire from a touch–screen pad. Participants reported the duration they spent outdoor light on a typical day in summer and winter (Supplementary Methods). Time spent in outdoor light in summer was moderately correlated with time in winter (Pearson’s *r* = 0.66, *P* < 0.001), which suggested time spent in outdoor light in summer and winter were not correlated perfectly. Therefore, we respectively examined the association of time spent in outdoor light in summer and in winter with the risk of depression [18]. We also calculated the average time by dividing the sum of time spent in outdoor light in winter and summer by 2 to uniform the standard of outdoor light exposure.

### Assessment of polygenic risk score (PRS)

A PRS for depression was calculated to evaluate the accumulating effect of depression genetic predisposition. PRS was built according to the meta-analysis of GWAS, which contained genome-wide association studies about the participants of European descent [19]; thus, only Whites from the UK Biobank were included in our study. The posterior effect sizes of nucleotide polymorphisms (SNPs) on depression were inferred using a PRS-CS, which uses Bayesian regression to place continuous shrinkage priors on SNP effect sizes from GWAS summary statistics [20]. PRS-CS incorporates an external LD reference panel to model local LD patterns and updates effect sizes jointly within LD blocks, allowing accommodation of diverse genetic architectures and avoiding decisions related to pruning and GWAS threshold selection. The weights were estimated using the PRS-CS method with default parameters and 1000 Genomes European as the LD reference panel, based on GWAS summary statistics for the major depressive disorder which were obtained from the Genetic Epidemiology Research on Adult Health and Aging (GERA) with a sample size of 61,847 (7892 cases and 53,955 controls). We retained autosomal 1,065,182 SNPs available in UK Biobank. A weighted PRS was calculated by the sum of the number of risk alleles of each SNP weighted by the risk estimate (β coefficient, namely the per-allele log odds ratio related to SNPn obtained from previous GWAS study) for depression across all available SNPs (*N* = 1,065,182) in the UK Biobank, which was produced using the PLINK “–score” command [21]. After conducting Z–standardization of PRS, all White participants were divided into three categories (based on the tertile distribution: low tertile, intermediate tertile, and high tertile of genetic risk).

### Ascertainment of depression

In terms of the information on hospital inpatient records and diagnoses (from the Hospital Episode Statistics for England, the Scottish Morbidity Record data for Scotland, and the Patient Episode Database for Wales) of depression, we identified the data on depression using the International Classification of Diseases (version10 [ICD-10] code: F32 - F34, F38 and F39). Follow-up was terminated until participants developed depression, death, or the study endpoint (September 30, 2021). More details on the hospital inpatient records are distributed in the Supplementary Methods.

### Covariates

Demographic characteristics included individuals’ age, gender, education level, and the Townsend deprivation index. Lifestyle characteristics included smoking status, drinking status, total physical activity, sleep duration, and body mass index (BMI), which could reflect individuals’ living habits. History of the disease (hypertension, hyperglycemia, fracture history in the past 5 years, and hearing loss) may reflect individuals’ long-term health condition and daily behavioral capacity. Vitamin D supplements and the use of sun/ultraviolet (UV) radiation protection may also reveal individuals’ habits of outdoor light. Air pollutants (PM_2.5_) may also play an important role in individuals’ travel and mental health. Among these covariates, the Townsend deprivation index was assigned based on postcode as a continuous measure, where a higher index indicates more deprivation. Total physical activity was measured as the sum of minutes spent walking per week and minutes engaged in moderate or vigorous activity per week during the past four weeks. Hypertension was defined as (1) hospital inpatient records (ICD-10 codes I10, I15, O10); (2) or self-reported cases; (3) or blood pressure ≥140/90 mmHg; (4) or taking anti-hypertensive therapy. Hyperglycemia was defined as (1) fasting blood glucose levels >110 mg/dL; (2) or taking anti-diabetic therapy; (3) or hospital inpatient records of diabetes (ICD-10 codes E10–E14); (4) or self-reported cases of diabetes.

### Statistical analyses

According to whether participants developed depression, we compared the characteristics of continuous variables and categorical variables at baseline using a *t-*test, Mann–Whitney *U*-test, and chi-squared test, respectively.

To get the completed data, multiple imputations by five replications and chained equations method were applied to impute the missing values for covariates. Dose-response association between time spent in outdoor light and the risk of depression was examined using the restricted cubic spline with five knots located at the 5th, 27.5th, 50th, 72.5th, and 95th percentiles [22]. We also tested the linearity or nonlinearity based on the Wald test [23]. If the null hypothesis was rejected (*P* < 0.05), it will show that a nonlinear relationship between time spent in outdoor light and the risk of depression is observed.

Incidence rates (IRs) per 1000 person-years were respectively calculated in terms of the category of PRS and time spent in outdoor light. The hazard ratios (HRs) and 95% confidence intervals (CIs) of depression risk concerning time spent in outdoor light and genetic risk were estimated via Cox proportional hazards regression models. The proportional hazard assumption was checked by tests based on Schoenfeld residuals, and the results indicated that the assumptions had not been violated. The demographic characteristics (age [continuous], gender, education level [college or university degree, upper secondary, lower secondary, vocational, and other], Townsend deprivation index [continuous]), lifestyle characteristics (smoking status [never, previous, and current], drinking status [never, previous, and current], total physical activity [MET-min/week, continuous], sleep duration [h/day, continuous], and BMI [continuous]), the history of the disease (hypertension, hyperglycemia, fracture history in past five years, and hearing loss), vitamin D supplement, use of sun/UV protection, PM_2.5_ and PRS were defined as potential confounders, which were adjusted in the Cox proportional hazards regression models. The multiplicative interaction between depression genetic risk and time spent in outdoor light was tested by containing these two exposure variables and their cross-product terms in the Cox model. We estimated the 50th percentile differences (PDs) for the median time (in years) to incident depression via Laplace regression. We estimated the joint effects of time spent in outdoor light with genetic predisposition and the risk of depression, individuals with the optimal time spent in outdoor light and the lower tertile of PRS were defined as the reference groups.

Several sensitive analyses were carried out: (1) excluding missing values of covariates; (2) excluding individuals who suffered from incident depression in the first 2-year duration follow-up to eliminate the effect of potential reverse causality; (3) excluding individuals whose average time spent in outdoor light were over twofold of standard deviation; (4) further adjusting for antidepressant use; (5) we conducted stratified analysis by using the sun/UV protection to investigate the association between time spent in outdoor light and depression risk; (6) to examine if the relationship between time spent in outdoor light and depression risk was different depending on the use of vitamin D supplements, we conducted a stratified analysis based on the individuals who used or did not use vitamin D supplements; (7) further adjusting for UK Biobank assessment centers; (8) further adjusting for employment (working, retired, unemployment, other); (9) further adjusting for the living environment (urban or rural areas); (10) individuals with less than 1 h of outdoor light in winter were divided into a separate group; (11) including participants spent extreme hours in outdoor light.

All tests were two-sided, and statistical significance was considered *P* < 0.05. Statistical analyses were performed in R V.1.4.

## Results

### Baseline characteristics of the study population

A total of 380,976 participants were included in our study, of which 13,636 (prevalence = 3.58%) depression cases were diagnosed during a median follow-up of 12.57 years (interquartile range: 11.81 to 13.28 years). Table 1 showed the comparison of baseline characteristics between depression patients and depression-free individuals. Compared to depression-free participants, those with incident depression tend to be older, female, current smokers, never or rarely used sun/UV protection, and more likely to have low education level, higher BMI, less sleep duration, higher PM_2.5_ exposure, and higher genetic risk (*P* < 0.05). Obviously, individuals with depression tend to have longer average time spent in outdoor light in comparison with depression-free individuals (*P* < 0.001).

**Table 1:** Baseline characteristics of participants.

Characteristics	No depression (*N* = 367,340)	Incident depression (*N* = 13,636)	*P* value
Age	56.81 ± 8.03	57.11 ± 8.19	<0.001
Gender			<0.001
Female	193,711 (52.7)	8416 (61.7)	
Male	173,629 (47.3)	5220 (38.3)	
Townsend deprivation index, mean (SD)	−1.61 ± 2.90	−0.83 ± 3.26	<0.001
Education			<0.001
College or University degree	120,690 (32.9)	3174 (23.3)	
Upper secondary	42,254 (11.5)	1385 (10.2)	
Lower secondary	101,595 (27.7)	4015 (29.4)	
Vocational	24,563 (6.7)	1042 (7.6)	
Other	78,238 (21.2)	4020 (29.5)	
Smoking status			<0.001
Never	204,655 (55.7)	6298 (46.2)	
Previous	128,179 (34.9)	5113 (37.5)	
Current	34,506 (9.4)	2225 (16.3)	
Drinking status			<0.001
Never	12,113 (3.3)	591 (4.3)	
Previous	10,908 (3.0)	881 (6.5)	
Current	344,319 (93.7)	12,164 (89.2)	
Body mass index (kg/m^2^), mean (SD)	27.3 ± 4.62	28.5 ± 5.48	<0.001
Total physical activity, MET-min/week, mean (SD)	681.3 ± 885.1	684.6 ± 945.4	0.681
Sleep duration, hours/day, mean (SD)	7.16 ± 1.04	7.11 ± 1.37	<0.001
PM_2.5_, ug/m^3^, mean (SD)	9.93 ± 1.04	10.07 ± 1.06	<0.001
Vitamin D supplement			<0.001
No	353,470 (96.2)	13,017 (95.5)	
Yes	13,870 (3.8)	619 (4.5)	
Use of sun/UV protection			<0.001
No or occasionally	153,755 (41.9)	5902 (43.3)	
Yes	211,910 (57.7)	7601 (55.7)	
Do not go out in the sunshine	1675 (0.4)	133 (1.0)	
Fracture history			<0.001
No	333,897 (90.9)	11,939 (87.6)	
Yes	33,443 (9.1)	1697 (12.4)	
Hearing loss			<0.001
No	232,799 (63.4)	7443 (54.6)	
Yes	134,541 (36.6)	6193 (45.4)	
Hypertension			0.012
No	189,143 (51.5)	6871 (50.4)	
Yes	178,197 (48.5)	6765 (49.6)	
Hyperglycemia			<0.001
No	337,804 (92.0)	12,117 (88.9)	
Yes	29,536 (8.0)	1519 (11.1)	
Genetic risk category			<0.001
Low	121,624 (33.1)	4097 (30.0)	
Intermediate	121,116 (33.0)	4607 (33.8)	
High	124,600 (33.9)	4932 (36.2)	
Time spent in outdoor light, hours/day, mean (SD)			
Summer	3.73 ± 2.28	3.87 ± 2.39	<0.001
Winter	1.79 ± 1.64	1.87 ± 1.70	<0.001
Average	2.76 ± 1.79	2.87 ± 1.84	<0.001

Data are *n* (%) and mean (SD). *P* values are derived using a *t*-test, Mann–Whitney *U*-test, and chi-squared test.

*SD* standard deviation, *UV* ultraviolet radiation.

### Independent association of time spent in outdoor light and genetic risk with incident depression

**Fig. 1:**
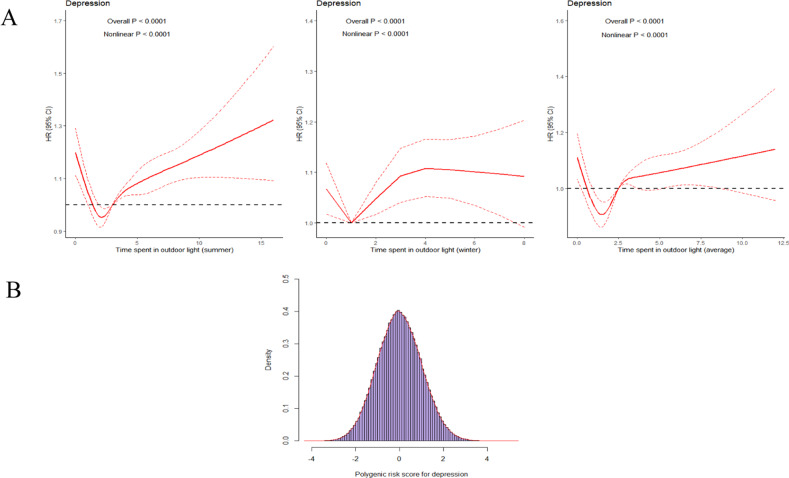
Risk of incident depression according to time spent in outdoor light and genetic risk. **A** The correlation between time spent in outdoor light and incident depression during follow-up; **B** Distribution of polygenic risk score by incident depression. HR hazard ratio, 95% CI 95% confidence interval. Adjusted for age, gender, education, Townsend deprivation index, smoking status, drinking status, body mass index, total physical activity, sleep duration, fracture history, vitamin D supplement, hearing loss, use of sun/UV protection, PM_2.5_, hypertension, hyperglycemia, and genetic risk.

A nonlinear (J-shaped) relationship was observed for time spent in outdoor light (as a continuous variable) and incident depression risk with restricted cubic splines models. For outdoor light, a significantly increased risk of low exposure was observed, but an increased risk of higher exposure was relatively slow. The association between time spent in outdoor light and depression risk presented a nonlinear trend (*P* < 0.05). Individuals with 1.5 h/day on average, 1 h/day in winter, and 2 h/day in summer respectively showed the lowest risk of depression. With those participants as a reference, an elevated risk of depression was significantly shown regardless of the groups below or above the reference value (Fig. 1A). In comparison with individuals with 1.5 h/day outdoor light on average, individuals below 1.5 h/day (HR = 1.09; 95% CI: 1.02–1.16) and above 1.5 h/day (HR = 1.13; 95% CI: 1.07–1.20) time spent in outdoor light had an increased depression risk. Furthermore, in comparison with the individuals who received 2 h/day of outdoor light in summer, individuals with below 2 h/day (HR = 1.12; 95% CI: 1.05–1.19) and above 2 h/day (HR = 1.11; 95% CI: 1.06–1.16) outdoor light were prone to having an elevated depression risk. In comparison with individuals with 1 h/day outdoor light in winter, individuals with below 1 h/day outdoor light were significantly associated with a 7% elevated depression risk (HR = 1.07; 95% CI: 1.02–1.13), and individuals with above 1 h/day outdoor light was significantly associated with a 9% elevated risk of depression (HR = 1.09; 95% CI: 1.04–1.13, Table 2).

**Table 2:** Hazard ratios (HRs) and 50th percentile differences (PDs, in years) of incident depression.

Time spent in outdoor light	Events/total	Incidence rate per 1000 person-year	Model 1		Model 2		E Value^c^
			HR^a^ /50th PD^b^ (95% CI)	*P* Value	HR^a^ /50th PD^b^ (95% CI)	*P* Value	
Summer							
2 h	2645/83466	2.59 (2.49, 2.69)	1 (Ref.)		1 (Ref.)		NA
			0 (Ref.)		0 (Ref.)		
Below 2 h	1798/49841	2.95 (2.82, 3.09)	1.14 (1.08, 1.21)^a^	<0.001	1.12 (1.05, 1.19)^a^	<0.001	1.49
			−0.65 (−0.95, −0.35)^b^	<0.001	−0.54 (−0.84, −0.24)^b^	<0.001	
Above 2 h	9193/247669	3.06 (3.00, 3.12)	1.19 (1.14, 1.24)^a^	<0.001	1.11 (1.06, 1.16)^a^	<0.001	1.46
			−0.87 (−1.08, −0.65)^b^	<0.001	−0.49 (−0.71, −0.27)^b^	<0.001	
Winter							
1 h	4133/127792	2.64 (2.56, 2.72)	1 (Ref.)		1 (Ref.)		NA
			0 (Ref.)		0 (Ref.)		
Below 1 h	2676/72906	3.01 (2.89, 3.12)	1.14 (1.08, 1.20)^a^	<0.001	1.07 (1.02, 1.13)^a^	0.005	1.34
			−0.63 (−0.87, −0.38)^b^	<0.001	−0.38 (−0.62, −0.14)^b^	0.002	
Above 1 h	6827/180278	3.13 (3.05, 3.20)	1.19 (1.14, 1.23)^a^	<0.001	1.09 (1.04, 1.13)^a^	<0.001	1.40
			−0.87 (−1.06, −0.67)^b^	<0.001	−0.40 (−0.59, −0.20)^b^	<0.001	
Average							
1.5 h	1539/50589	2.48 (2.36, 2.60)	1 (Ref.)		1 (Ref.)		NA
			0 (Ref.)		0 (Ref.)		
Below 1.5 h	2469/71933	2.80 (2.69, 2.91)	1.13 (1.06, 1.21)^a^	<0.001	1.09 (1.02, 1.16)^a^	0.012	1.40
			−0.61 (−0.93, −0.29)^b^	<0.001	−0.46 (−0.78, −0.14)^b^	0.005	
Above 1.5 h	9628/258454	3.07 (3.01, 3.13)	1.24 (1.18, 1.31)^a^	<0.001	1.13 (1.07, 1.20)^a^	<0.001	1.51
			−1.11 (−1.38, −0.84)^b^	<0.001	−0.63 (−0.90, −0.35)^b^	<0.001	

Model 1 was unadjusted; Model 2 adjusted for age, gender, education, Townsend deprivation index, smoking status, drinking status, body mass index, total physical activity, sleep duration, fracture history, vitamin D supplement, hearing loss, use of sun/UV protection, PM2.5, hypertension, hyperglycemia, and genetic risk.

*HR* hazard ratio, *50th PD* the difference in the median time (in years) until the first 50% of the participants, *95% CI* 95% confidence interval.

^a^Hazard ratios (HRs) and 95% confidence intervals (CIs) of incident depression by Cox models.

^b^The 50th percentile difference (PDs, years) and 95% CIs of incident depression by Laplace regression models.

^c^The E value is the minimum strength of association, on the risk ratio scale, that an unmeasured confounder would need to have with both the treatment and outcome, conditional on the measured covariat to explain away a treatment-outcome association.

Laplace regression analyses reported that in comparison of individuals with 2 h/days spent in outdoor light in summer, people with below 2 h/days outdoor light developed incident depression 0.54 (50th PD, 95% CI: −0.84, −0.24) years earlier, and the time to incident depression was shortened by 0.49 (50th PD, 95% CI: −0.71, −0.27) years among individuals who spent >2 h/day outdoor light. In the analyses of winter or average time spent in outdoor light, individuals with below or above the time of reference standard also developed incident depression slightly earlier (Table 2).

PRS for depression was normally distributed (Fig. 1B), and almost close to two-thirds of individuals with above 1.5 h/day outdoor light on average (64.1%, Supplementary Fig. S2). The restricted spline curve in Supplementary Fig. S3 presented a linear trend and dose-response relationship between genetic predisposition and the risk of incident depression (*P* for nonlinear = 0.307). After full adjustment for potential confounders, the IRs per 1000 person-years of depression was 2.67 (2.59–2.76), 3.01 (2.92–3.10), and 3.13 (3.05–3.22) in individuals with low tertile, intermediate tertile, and high tertile of PRS, respectively. In multi-adjusted Cox regression models, the HRs and 95% CIs of depression were 1.12 (1.08–1.17) for individuals with an intermediate tertile of PRS and 1.16 (1.11–1.21) for individuals with a high tertile of PRS in comparison with individuals with a low tertile of PRS (Table 3).

**Table 3:** Risk of incident depression according to genetic risk.

Genetic risk	Events/total	Incidence rate per 1000 person-year	Model 1^a^	Model 2^b^	Model 3^c^
			HR (95% CI)	HR (95% CI)	HR (95% CI)
Continuous	13636/380976	2.94 (2.89, 2.99)	1.08 (1.06, 1.09)	1.07 (1.05, 1.09)	1.07 (1.05, 1.09)
Categorical					
Low	4097/125721	2.67 (2.59, 2.76)	1 (Ref.)	1 (Ref.)	1 (Ref.)
Intermediate	4607/125723	3.01 (2.92, 3.10)	1.13 (1.08, 1.18)	1.13 (1.08, 1.17)	1.12 (1.08, 1.17)
High	4932/129532	3.13 (3.05, 3.22)	1.17 (1.12, 1.22)	1.16 (1.11, 1.21)	1.16 (1.11, 1.21)
*P* for trend			<0.001	<0.001	<0.001

*HR* hazard ratio, *95% CI* 95% confidence interval.

^a^Model 1: Adjusted for age, sex, education, Townsend deprivation index.

^b^Model 2: Adjusted for age, sex, education, Townsend deprivation index, smoking status, drinking status, body mass index, total physical activity, and sleep duration.

^c^Model 3: Adjusted for age, sex, education, Townsend deprivation index, smoking status, drinking status, body mass index, total physical activity,sleep duration, fracture history, vitamin D supplement, hearing loss, use of sun/UV protection, PM2.5, hypertension, hyperglycemia, and time spent in outdoor light.

### The joint effect of time spent in outdoor light and genetic predisposition on depression risk

**Fig. 2:**
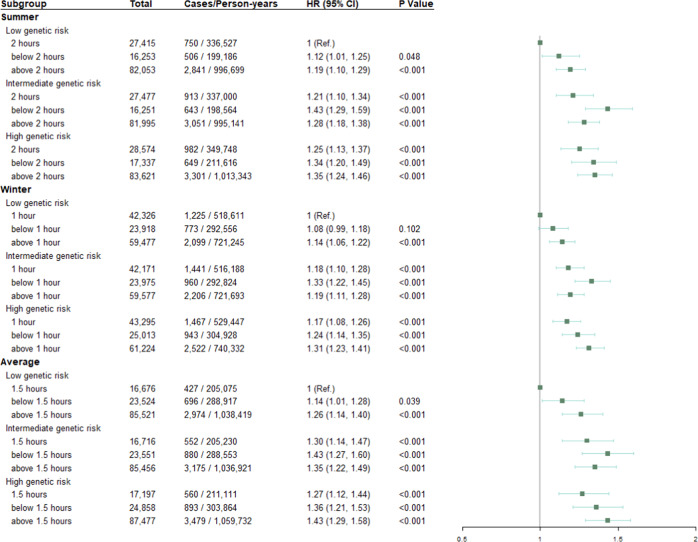
Risk of incident depression according to genetic risk and time spent in outdoor light. HR hazard ratio, 95% CI 95% confidence interval. Adjusted for age, gender, education, Townsend deprivation index, smoking status, drinking status, body mass index, total physical activity, sleep duration, fracture history, vitamin D supplement, hearing loss, use of sun/UV protection, PM_2.5_, hypertension, and hyperglycemia.

Figure 2 shows the association between depression risk and the joint association between time spent in outdoor light in summer and genetic risk. In the joint effect analysis, in comparison of those with low tertile of PRS and 2 h/day outdoor light in summer, a 34% higher risk of depression was observed (HR = 1.34; 95% CI: 1.20–1.49) in those with high tertile of PRS and below 2 h/day outdoor light; and 35% higher risk of depression were observed (HR = 1.35; 95% CI: 1.24–1.46) in those with high tertile of PRS and above 2 h/day outdoor light. Similarly, in comparison of those with low tertile of PRS and 1 h/day outdoor light in winter, high tertile of PRS and below 1 h/day outdoor light was associated with a 24% higher risk of depression (HR = 1.24; 95% CI: 1.14–1.35); and high tertile of PRS and above 1 h/day outdoor light was associated with a 31% higher risk of depression (HR = 1.31; 95% CI: 1.23–1.41). Of individuals with a high tertile of PRS and below 1.5 h/day outdoor light on average, 36% increased risk of developed depression vs these individuals with low tertile of PRS and 1.5 h/day outdoor light (HR = 1.36; 95% CI, 1.21–1.53); and of individuals with a high tertile of PRS and above 1.5 h/day outdoor light, 43% increased risk of developed depression (HR = 1.43; 95% CI, 1.29–1.58). Although among people with low genetic risk, compared with 1.5 h/day outdoor light on average, the increased risk of depression associated with below 1.5 h/day and above 1.5 h/day outdoor light was respectively 1.14 (95% CI, 1.01–1.28) and 1.26 (95% CI, 1.14–1.40).

The significant interactions were respectively observed between intermediate genetic risk and longer time spent in outdoor light (*P*_average_ for interaction = 0.005; *P*_summer_ for interaction = 0.024; *P*_winter_ for interaction = 0.013) (Supplementary Table S1).

### Additional analyses

The following analysis could evaluate of stability of our study results, we obtained similar results. (1) We conducted stratified analysis by age (Supplementary Table S2) and sex (Supplementary Table S3). (2) We repeated the analysis by excluding the missing data on covariables. (Supplementary Table S4). (3) Individuals who developed incident depression within the first 2-years during follow-up were excluded (Supplementary Table S5). (4) We repeated the analysis by excluding individuals whose average time spent in outdoor light was over twofold the standard deviation (Supplementary Table S6). (5) We further adjusted for antidepressant use (Supplementary Table S7). (6) We conducted stratified analysis by using sun/UV protection (Supplementary Table S8). We found among the individuals who used sun/UV protection, the association between below optimal time spent in outdoor light (<2 h/day in summer, spent <1 h/day in winter, and spent 1.5 h/day on average) and the risk of depression was statistically insignificant. (7) Supplementary Table S9 shows the stratified analysis based on individuals with or without vitamin D supplements. We found among individuals who used vitamin D supplements, the association between time spent in outdoor light and the risk of depression was statistically insignificant. (8) We further adjusted for UK Biobank assessment centers (Supplementary Tables S10, S11). (9) We further adjusted for employment (Supplementary Table S12). (10) We further adjusted for the living environment (Supplementary Table S13). (11) The dose-response association between time spent in outdoor light in winter and depression risk after dividing individuals spent less than 1 h into a group (Supplementary Fig. S4). (12) We included participants with extreme hours in outdoor light, 2 h/day outdoor light in summer, 1 h/day in winter, and 1.5 h/day on average were respectively still associated with the lowest risk of depression (Supplementary Fig. S5). Below and above the optimal time were still associated with the increased risk of depression (Supplementary Table S14).

## Discussion

In UK Biobank, we found that (1) a nonlinear trend (J-shaped relationship) was observed between time spent in outdoor light and depression risk, an average of 1.5 h/day outdoor light was related to the lowest risk of depression, the optimal time to minimize the risk of depression was respectively at 2 h/day in summer and 1 h/day in winter; (2) spending shorter and longer time in outdoor light might advance the onset of depression by about 0.5 years compared with optimal time; (3) after combining the depression genetic risk and time spent in outdoor light, high tertile of PRS and below or above average 1.5 h/day outdoor light were both associated with 1.40-fold elevated depression risk in comparison with those with a low tertile of PRS of depression and optimum time.

Evaluation of genetic susceptibility to diseases in the form of PRS was derived from risk alleles based on the largest available GWAS of those diseases [17, 24]. PRS has been shown to be a much more effective instrument than any single risk gene [25]. This is because PRS was calculated as the sum of risk alleles weighted by their individual severally estimated effect sizes [26]. Previous evidence has shown the heritability of depression to be 30–40% [27]. One study from the Erasmus Rucphen Family cohort found PRS to be significantly closely related to the incidence of depression ascertained from various measure methods [28]. Higher PRS for depression was associated with an elevated risk of depression, and the fact that the estimated genetic association with depression in the UK Biobank was higher in patients diagnosed with depression by a physician was included [29]. In this study, a high tertile of PRS for depression was associated with a more than 1.5-fold elevated risk of depression over those with a low tertile as calculated from previous GWAS data. Genes and environments do not operate independently, and gene-environment interaction may influence the results [30]. There was a significant interaction in the stratified analyses between PRS and outdoor light time. It suggests that genetic risk and time spent in outdoor light mutually influence to some extent.

Several studies have reported an association between time spent in outdoor light and depression [8, 9]. One study showed that depression patients who lived in rooms with east-facing windows or equivalent had hospital stays about 4 days shorter than those who lived with west-facing rooms without direct outdoor light [9]. However, another cross-sectional study suggested that outdoor light may have a bimodal impact on the risk of depression such that short-term exposure to outdoor light may increase the risk of depression but long-term exposure may decrease it [10]. In our study, we found that longer and shorter times spent in outdoor light were both associated with an increased risk of depression. J-shaped nonlinear associations between time spent in outdoor light and risk of depression were observed for both summer and winter. Several previous studies suggested that bright light may be significantly closely associated with a decreased risk of depression no matter the duration of light exposure [31–34]. However, these studies tend to focus on indoor lighting (e.g., the workplace) or fluorescent lighting rather than outdoor daylight. The difference between our findings and prior studies may be due to the differences in wavelength.

There might be mechanisms underlying the association between time spent in outdoor light and the risk of depression. First, prolonged exposure to outdoor light has been found to be related to an elevated risk of depression, which may be attributable to the production of reduced melatonin. Long-term exposure to UV can affect an individual’s psychological and neuroendocrine parameters. One study [35] reported that 53 participants underwent 6 rounds of UVA exposure in 3 weeks. After the first UVA treatment, the volunteers experienced a significant decrease in melatonin. Previous studies have found that UVA radiation has an extremely important effect on pineal melatonin production [36, 37]. Reduced melatonin secretion can affect a person’s circadian rhythms [38]. Circadian rhythm disruption underlies the pathophysiology of psychiatric disorders, especially depression [39]. In addition, long-time UV exposure can induce the production of IL-1β and IL-6 [40]. IL-6, a type of pro-inflammatory cell, is involved in the pathophysiology of major depressive disorder in the brain [41, 42]. Second, the rate of serotonin production in the brain is correlated with the duration of sunlight exposure [43]. A lack of outdoor light can influence the production of serotonin, while changes in serotonin may cause seasonal variations in mood [44]. A lack of outdoor light exposure may reduce vitamin D production, which has been reported to be associated with the pathogenesis and seasonality of depression [45]. Many people only rarely spend time in outdoor light because of high stress or long work hours. Urbanization and income levels are also important trigger factors. This issue merits further exploration.

During 12.6 years of follow-up, shorter and longer periods spent in outdoor light was associated with a 7–13% elevated risk of depression. We calculated the E value to evaluate the robustness of the result. The E value we calculated was higher than the effective value of time spent in outdoor light on outcomes in previous studies [18, 46], which indicates that our results are relatively robust. Given that genetic and environmental factors may contribute to the risk of depression collectively, we investigated the interaction and joint association of outdoor light and the PRS of depression. We found optimum outdoor light time was associated with a lower risk of incident depression, genetically predetermined elevated risk of depression might be counteracted to some extent by optimum time spent in outdoor light. Future studies are required to explore whether other factors influence the association between time spent in outdoor light and the risk of depression, for example, physical activity.

To our knowledge, no attention has been paid to investigating the combination of time spent in outdoor light and PRS associated with depression risk. In this study, we explored the nonlinear trend between time spent in outdoor light and the risk of depression based on the biggest advantage of a large-scale sample. The joint effects of time spent in outdoor light and genetic susceptibility with the risk of depression were also investigated by using the information on a genome-wide depression. However, the present work has some limitations. First, time spent in outdoor light was self-reported at baseline, so it may involve recall bias. Second, the participants in this study were all whites, and the intensity and duration of outdoor light varied by region. The applicability of these findings to other regions and other ethnic groups remains to be verified. Third, there are many psychosocial and environmental factors affecting the onset of depression. Given that many people often work in an office and overwork, they do not spend time in outdoor light, working conditions and workload can easily affect the probability of suffering from depression. Besides, living environment and socioeconomic status also play a vital role in the relationship between outdoor light time and depression risk. Individuals living in rural areas may be prone to spending time in outdoor light. Moreover, they often have lower socioeconomic status in comparison with individuals living the urban areas. Despite including many potential confounders, there are still many not accounted for, for example, the workloads of participants, sufficient details about their jobs, and the temperature and intensity of outdoor light. Fourth, in our analyses, the diagnosis of depression was based on patients’ hospital admission records, which may involve some underestimation of the incidence of depression. Participants from the UK Biobank cohort tended to be more health-conscious and have healthier lifestyles than nonparticipants, which may also have caused us to underestimate the incidence of depression. Future studies with different samples and more precise study designs are required to replicate our findings.

## Conclusions

Time spent in outdoor light and genetic risk were independently associated with incident depression. The optimum time spent in outdoor light—including 2 h/day in summer, 1 h/day in winter, and 1.5 h/day on average—may attenuate depression risk, which suggests the benefits of spending optimal time spent in outdoor light in entire populations, independent of depression genetic predisposition.

## Supplementary information

supplemental materials

## Data Availability

Data from the UK Biobank cannot be shared publicly, however, data were available from the UK Biobank Institutional Data Access/Ethics Committee (contact via http://www.ukbiobank.ac.uk/ or contact by email at access@ukbiobank.ac.uk) for researchers who meet the criteria for access to confidential data.

## References

[1] Bromet E, Andrade LH, Hwang I, Sampson NA, Alonso J, de Girolamo G (2011). Cross-national epidemiology of DSM-IV major depressive episode. BMC Med.

[2] Collaborators CMD (2021). Global prevalence and burden of depressive and anxiety disorders in 204 countries and territories in 2020 due to the COVID-19 pandemic. Lancet.

[3] Malhi GS, Mann JJ (2018). Depression. Lancet.

[4] Meng R, Yu C, Liu N, He M, Lv J, Guo Y (2020). Association of depression with all-cause and cardiovascular disease mortality among adults in China. JAMA Netw Open.

[5] Gilman SE, Sucha E, Kingsbury M, Horton NJ, Murphy JM, Colman I (2017). Depression and mortality in a longitudinal study: 1952-2011. Can Med Assoc J.

[6] Anglin RE, Samaan Z, Walter SD, McDonald SD (2013). Vitamin D deficiency and depression in adults: systematic review and meta-analysis. Brit J Psychiatry.

[7] Black LJ, Seamans KM, Cashman KD, Kiely M (2012). An updated systematic review and meta-analysis of the efficacy of vitamin D food fortification. J Nutr.

[8] Kent ST, McClure LA, Crosson WL, Arnett DK, Wadley VG, Sathiakumar N (2009). Effect of sunlight exposure on cognitive function among depressed and non-depressed participants: a REGARDS cross-sectional study. Environ Health Glob.

[9] Benedetti F, Colombo C, Barbini B, Campori E, Smeraldi E (2001). Morning sunlight reduces length of hospitalization in bipolar depression. J Affect Disorders.

[10] Son J, Shin J (2021). Bimodal effects of sunlight on major depressive disorder. Compr Psychiatry.

[11] Keller J, Gomez R, Williams G, Lembke A, Lazzeroni L, Murphy GJ (2017). HPA axis in major depression: cortisol, clinical symptomatology and genetic variation predict cognition. Mol Psychiatry.

[12] Feng XL, Che HL, Ning X, Ba XY, Li J, Zhang JF (2019). Direct sunlight exposure reduces hair cortisol levels in rhesus monkeys (Macaca mulatta). Zool Res.

[13] Li X, Luo Z, Gu C, Hall LS, McIntosh AM, Zeng Y (2018). Common variants on 6q16.2, 12q24.31 and 16p13.3 are associated with major depressive disorder. Neuropsychopharmacol.

[14] Huo YX, Huang L, Zhang DF, Yao YG, Fang YR, Zhang C (2016). Identification of SLC25A37 as a major depressive disorder risk gene. J Psychiatry Res.

[15] Rice F, Harold G, Thapar A (2002). The genetic aetiology of childhood depression: a review. J Child Psychol Psychiatry.

[16] Hyde CL, Nagle MW, Tian C, Chen X, Paciga SA, Wendland JR (2016). Identification of 15 genetic loci associated with risk of major depression in individuals of European descent. Nat Genet.

[17] Wray NR, Ripke S, Mattheisen M, Trzaskowski M, Byrne EM, Abdellaoui A (2018). Genome-wide association analyses identify 44 risk variants and refine the genetic architecture of major depression. Nat Genet.

[18] Ma LZ, Ma YH, Ou YN, Chen SD, Yang L, Dong Q (2022). Time spent in outdoor light is associated with the risk of dementia: a prospective cohort study of 362094 participants. BMC Med.

[19] Zhu Z, Zheng Z, Zhang F, Wu Y, Trzaskowski M, Maier R (2018). Causal associations between risk factors and common diseases inferred from GWAS summary data. Nat Commun.

[20] Ge T, Chen CY, Ni Y, Feng YA, Smoller JW (2019). Polygenic prediction via Bayesian regression and continuous shrinkage priors. Nat Commun.

[21] Zhang Y, Yang R, Dove A, Li X, Yang H, Li S (2022). Healthy lifestyle counteracts the risk effect of genetic factors on incident gout: a large population-based longitudinal study. BMC Med.

[22] Nunez E, Steyerberg EW, Nunez J (2011). [Regression modeling strategies]. Rev Esp Cardiol.

[23] Desquilbet L, Mariotti F (2010). Dose-response analyses using restricted cubic spline functions in public health research. Stat Med.

[24] Ripke S (2014). NBMC. Biological insights from 108 schizophrenia-associated genetic loci. Nature.

[25] Colodro-Conde L, Couvy-Duchesne B, Zhu G, Coventry WL, Byrne EM, Gordon S (2018). A direct test of the diathesis-stress model for depression. Mol Psychiatry.

[26] Wray NR, Lee SH, Mehta D, Vinkhuyzen AA, Dudbridge F, Middeldorp CM (2014). Research review: polygenic methods and their application to psychiatric traits. J Child Psychol Psychiatry.

[27] Sullivan PF, Neale MC, Kendler KS (2000). Genetic epidemiology of major depression: review and meta-analysis. Am J Psychiatry.

[28] Demirkan A, Penninx BW, Hek K, Wray NR, Amin N, Aulchenko YS (2011). Genetic risk profiles for depression and anxiety in adult and elderly cohorts. Mol Psychiatry.

[29] Mitchell BL, Thorp JG, Wu Y, Campos AI, Nyholt DR, Gordon SD (2021). Polygenic risk scores derived from varying definitions of depression and risk of depression. JAMA Psychiatry.

[30] Akimova ET, Breen R, Brazel DM, Mills MC (2021). Gene-environment dependencies lead to collider bias in models with polygenic scores. Sci Rep.

[31] Avery DH, Kizer D, Bolte MA, Hellekson C (2001). Bright light therapy of subsyndromal seasonal affective disorder in the workplace: morning vs. afternoon exposure. Acta Psychiatr Scand.

[32] Wirz-Justice A, Bucheli C, Graw P, Kielholz P, Fisch HU, Woggon B (1986). Light treatment of seasonal affective disorder in Switzerland. Acta Psychiatr Scand.

[33] Partonen T, Lonnqvist J (2000). Bright light improves vitality and alleviates distress in healthy people. J Affect Disorders.

[34] Tao L, Jiang R, Zhang K, Qian Z, Chen P, Lv Y (2020). Light therapy in non-seasonal depression: an update meta-analysis. Psychiat Res.

[35] Gambichler T, Bader A, Vojvodic M, Bechara FG, Sauermann K, Altmeyer P (2002). Impact of UVA exposure on psychological parameters and circulating serotonin and melatonin. BMC Dermatol.

[36] Brainard GC, Podolin PL, Leivy SW, Rollag MD, Cole C, Barker FM (1986). Near-ultraviolet radiation suppresses pineal melatonin content. Endocrinology.

[37] Zawilska JB, Rosiak J, Nowak JZ (1999). Effects of near-ultraviolet (UV-A) light on melatonin biosynthesis in vertebrate pineal gland. Biol Signals Recept.

[38] Xie Z, Chen F, Li WA, Geng X, Li C, Meng X (2017). A review of sleep disorders and melatonin. Neurol Res.

[39] Satyanarayanan SK, Su H, Lin YW, Su KP (2018). Circadian rhythm and melatonin in the treatment of depression. Curr Pharm Design.

[40] Urbanski A, Schwarz T, Neuner P, Krutmann J, Kirnbauer R, Kock A (1990). Ultraviolet light induces increased circulating interleukin-6 in humans. J Invest Dermatol.

[41] Licinio J, Wong ML (1999). The role of inflammatory mediators in the biology of major depression: central nervous system cytokines modulate the biological substrate of depressive symptoms, regulate stress-responsive systems, and contribute to neurotoxicity and neuroprotection. Mol Psychiatry.

[42] Tsai SJ (2017). Effects of interleukin-1beta polymorphisms on brain function and behavior in healthy and psychiatric disease conditions. Cytokine Growth Factor Rev.

[43] Ji Y, Chen C, Xu G, Song J, Su H, Wang H (2023). Effects of sunshine duration on daily outpatient visits for depression in Suzhou, Anhui Province, China. Environ Sci Pollut Res Int.

[44] Lambert GW, Reid C, Kaye DM, Jennings GL, Esler MD (2002). Effect of sunlight and season on serotonin turnover in the brain. Lancet.

[45] Geng C, Shaikh AS, Han W, Chen D, Guo Y, Jiang P (2019). Vitamin D and depression: mechanisms, determination and application. Asia Pac J Clin Nutr.

[46] Kim SY, Bang M, Wee JH, Min C, Yoo DM, Han SM (2021). Short- and long-term exposure to air pollution and lack of sunlight are associated with an increased risk of depression: A nested case-control study using meteorological data and national sample cohort data. Sci Total Environ.

