# Supplementary material for: Association of time spent in outdoor light and genetic risk with the incidence of depression
Source: Transl Psychiatry. 2023 Feb 3;13:40. doi: 10.1038/s41398-023-02338-0 (PMC9898270; doi:10.1038/s41398-023-02338-0)
Supplement: Supplementary file 1 — supplemental materials [file 41398_2023_2338_MOESM1_ESM.docx]

**Supplement materials**

**Supplementary Methods**

**Collection of information on outdoor light**

Participants wrote a number using a touch–screen pad or selected integers that included “less than an hour a day”, “do not know”, or “prefer not to answer” in several options set in advance. If participants spent a lot of time outdoors, they should report the average time they spent outdoors. We excluded 30,232 participants who reported “do not know” or “prefer not to answer”. And we redefined “less than an hour a day” as 0. We also excluded extreme values >16 hours on summer typical day and >8 hours on winter typical day (N = 4,647).(1) To unify outdoor light time standard, the duration reported in winter and summer was averaged.

**Source of hospital inpatient records of depression**

Participants joined the UK Biobank study from 2006 to 2010. Depression was ascertained using hospital inpatient records containing data on admissions and diagnoses obtained from the Hospital Episode Statistics for England, Scottish Morbidity Record data for Scotland, and the Patient Episode Database for Wales.

| Hospital inpatient records | ICD-10 code of depression |
| --- | --- |
| The Hospital Episode Statistics for England | F32, F33, F34, F38, F39 |
| The Scottish Morbidity Record data for Scotland | F32, F33, F34, F39 |
| The Patient Episode Database for Wales | F32, F33, F39 |

**Table S1. Interaction between the genetic risk and outdoor light time for depression.**

|  | HR (95% CI) | *P* value |
| --- | --- | --- |
| Summer ^a^ |  |  |
| Intermediate genetic risk × below 2 hours | 1.05 (0.90, 1.22) | 0.532 |
| Intermediate genetic risk × above 2 hours | 0.88 (0.79, 0.98) | 0.024 |
| High genetic risk × below 2 hours | 0.96 (0.82, 1.11) | 0.565 |
| High genetic risk × above 2 hours | 0.91 (0.81, 1.01) | 0.074 |
| Winter b |  |  |
| Intermediate genetic risk × below 1 hour | 1.04 (0.92, 1.18) | 0.515 |
| Intermediate genetic risk × above 1 hour | 0.88 (0.80, 0.97) | 0.013 |
| High genetic risk × below 1 hour | 0.98 (0.87, 1.11) | 0.806 |
| High genetic risk × above 1 hour | 0.99 (0.90, 1.09) | 0.874 |
| Average c |  |  |
| Intermediate genetic risk × below 1.5 hours | 0.97 (0.83, 1.14) | 0.724 |
| Intermediate genetic risk × above 1.5 hours | 0.82 (0.72, 0.94) | 0.005 |
| High genetic risk × below 1.5 hours | 0.94 (0.80, 1.11) | 0.482 |
| High genetic risk × above 1.5 hours | 0.89 (0.78, 1.02) | 0.094 |

Abbreviation: HR, hazard ratio; 95% CI, 95% confidence interval.

Shown are the interaction effects between intermediate or high genetic risk and different outdoor light time, ^a^ compared to low genetic risk and 2 hours in summer, ^b^ compared to low genetic risk and 1 hour in winter, ^c^ compared to low genetic risk and 1.5 hours average.

**Table S2. Hazard ratios (95% confidence interval) of incident depression according to genetic risk and outdoor light time stratified by age.**

| Subgroup | Age <60 | | Age ≥60 | |
| --- | --- | --- | --- | --- |
|  | HR (95% CI) | *P* Value | HR (95% CI) | *P* Value |
| Summer |  |  |  |  |
| Low genetic risk |  |  |  |  |
| 2 hours | 1 (Ref.) |  | 1 (Ref.) |  |
| below 2 hours | 1.07 (0.93, 1.23) | 0.324 | 1.24 (1.02, 1.50) | 0.033 |
| above 2 hours | 1.18 (1.06, 1.31) | 0.002 | 1.21 (1.06, 1.38) | 0.005 |
| Intermediate genetic risk |  |  |  |  |
| 2 hours | 1.19 (1.06, 1.34) | 0.004 | 1.25 (1.07, 1.47) | 0.006 |
| below 2 hours | 1.33 (1.17, 1.51) | <0.001 | 1.67 (1.40, 2.00) | <0.001 |
| above 2 hours | 1.22 (1.10, 1.35) | <0.001 | 1.34 (1.17, 1.52) | <0.001 |
| High genetic risk |  |  |  |  |
| 2 hours | 1.20 (1.06, 1.35) | 0.003 | 1.33 (1.14, 1.56) | <0.001 |
| below 2 hours | 1.22 (1.07, 1.40) | 0.002 | 1.60 (1.34, 1.91) | <0.001 |
| above 2 hours | 1.33 (1.20, 1.47) | <0.001 | 1.37 (1.20, 1.56) | <0.001 |
| Winter |  |  |  |  |
| Low genetic risk |  |  |  |  |
| 1 hour | 1 (Ref.) |  | 1 (Ref.) |  |
| below 1 hour | 1.06 (0.94, 1.18) | 0.364 | 1.14 (0.98, 1.31) | 0.084 |
| above 1 hour | 1.19 (1.08, 1.31) | <0.001 | 1.08 (0.97, 1.20) | 0.169 |
| Intermediate genetic risk |  |  |  |  |
| 1 hour | 1.21 (1.09, 1.33) | <0.001 | 1.15 (1.02, 1.29) | 0.019 |
| below 1 hour | 1.32 (1.19, 1.47) | <0.001 | 1.36 (1.19, 1.56) | <0.001 |
| above 1 hour | 1.14 (1.03, 1.26) | 0.009 | 1.21 (1.10, 1.35) | <0.001 |
| High genetic risk |  |  |  |  |
| 1 hour | 1.13 (1.02, 1.24) | 0.023 | 1.22 (1.09, 1.37) | 0.001 |
| below 1 hour | 1.23 (1.10, 1.37) | <0.001 | 1.27 (1.11, 1.46) | <0.001 |
| above 1 hour | 1.36 (1.24, 1.50) | <0.001 | 1.26 (1.14, 1.39) | <0.001 |
| Average |  |  |  |  |
| Low genetic risk |  |  |  |  |
| 1.5 hours | 1 (Ref.) |  | 1 (Ref.) |  |
| below 1.5 hours | 1.08 (0.93, 1.26) | 0.293 | 1.25 (1.02, 1.54) | 0.034 |
| above 1.5 hours | 1.23 (1.08, 1.40) | 0.002 | 1.32 (1.12, 1.56) | 0.001 |
| Intermediate genetic risk |  |  |  |  |
| 1.5 hours | 1.27 (1.09, 1.49) | 0.003 | 1.34 (1.09, 1.66) | 0.007 |
| below 1.5 hours | 1.33 (1.16, 1.54) | <0.001 | 1.65 (1.35, 2.01) | <0.001 |
| above 1.5 hours | 1.26 (1.11, 1.44) | <0.001 | 1.45 (1.23, 1.72) | <0.001 |
| High genetic risk |  |  |  |  |
| 1.5 hours | 1.23 (1.05, 1.44) | 0.012 | 1.35 (1.10, 1.67) | 0.005 |
| below 1.5 hours | 1.26 (1.09, 1.45) | 0.002 | 1.60 (1.31, 1.94) | <0.001 |
| above 1.5 hours | 1.38 (1.21, 1.57) | <0.001 | 1.51 (1.28, 1.78) | <0.001 |

Abbreviation: HR, hazard ratio; 95% CI, 95% confidence interval. Adjusted for sex, education, Townsend deprivation index, smoking status, drinking status, body mass index, total physical activity, sleep duration, fracture history, vitamin D supplement, hearing loss, use of sun/UV protection, PM_2.5_, hypertension, and hyperglycemia.

**Table S3. Hazard ratios (95% confidence interval) of incident depression according to genetic risk and outdoor light time stratified by sex.**

| Subgroup | Women | | Men | |
| --- | --- | --- | --- | --- |
|  | HR (95% CI) | *P* Value | HR (95% CI) | *P* Value |
| Summer |  |  |  |  |
| Low genetic risk |  |  |  |  |
| 2 hours | 1 (Ref.) |  | 1 (Ref.) |  |
| below 2 hours | 1.05 (0.91, 1.20) | 0.531 | 1.27 (1.05, 1.54) | 0.014 |
| above 2 hours | 1.14 (1.04, 1.26) | 0.008 | 1.27 (1.11, 1.46) | 0.001 |
| Intermediate genetic risk |  |  |  |  |
| 2 hours | 1.21 (1.07, 1.36) | 0.002 | 1.23 (1.03, 1.45) | 0.019 |
| below 2 hours | 1.39 (1.22, 1.58) | <0.001 | 1.50 (1.25, 1.80) | <0.001 |
| above 2 hours | 1.25 (1.13, 1.38) | <0.001 | 1.32 (1.15, 1.52) | <0.001 |
| High genetic risk |  |  |  |  |
| 2 hours | 1.21 (1.08, 1.36) | 0.001 | 1.31 (1.11, 1.55) | 0.001 |
| below 2 hours | 1.32 (1.16, 1.49) | <0.001 | 1.38 (1.15, 1.66) | 0.001 |
| above 2 hours | 1.31 (1.19, 1.44) | <0.001 | 1.41 (1.23, 1.62) | <0.001 |
| Winter |  |  |  |  |
| Low genetic risk |  |  |  |  |
| 1 hour | 1 (Ref.) |  | 1 (Ref.) |  |
| below 1 hour | 1.09 (0.98, 1.22) | 0.125 | 1.06 (0.90, 1.24) | 0.508 |
| above 1 hour | 1.16 (1.06, 1.27) | 0.002 | 1.10 (0.98, 1.23) | 0.123 |
| Intermediate genetic risk |  |  |  |  |
| 1 hour | 1.18 (1.07, 1.30) | 0.001 | 1.18 (1.04, 1.35) | 0.010 |
| below 1 hour | 1.33 (1.20, 1.48) | <0.001 | 1.33 (1.14, 1.55) | <0.001 |
| above 1 hour | 1.26 (1.15, 1.38) | <0.001 | 1.09 (0.97, 1.22) | 0.148 |
| High genetic risk |  |  |  |  |
| 1 hour | 1.18 (1.08, 1.30) | 0.001 | 1.14 (1.01, 1.29) | 0.048 |
| below 1 hour | 1.26 (1.14, 1.40) | <0.001 | 1.18 (1.01, 1.38) | 0.032 |
| above 1 hour | 1.35 (1.24, 1.48) | <0.001 | 1.25 (1.12, 1.40) | <0.001 |
| Average |  |  |  |  |
| Low genetic risk |  |  |  |  |
| 1.5 hours | 1 (Ref.) |  | 1 (Ref.) |  |
| below 1.5 hours | 1.11 (0.96, 1.29) | 0.159 | 1.19 (0.96, 1.47) | 0.119 |
| above 1.5 hours | 1.24 (1.09, 1.40) | 0.001 | 1.31 (1.10, 1.57) | 0.003 |
| Intermediate genetic risk |  |  |  |  |
| 1.5 hours | 1.28 (1.10, 1.50) | 0.001 | 1.32 (1.06, 1.65) | 0.014 |
| below 1.5 hours | 1.43 (1.24, 1.64) | <0.001 | 1.43 (1.16, 1.75) | 0.001 |
| above 1.5 hours | 1.34 (1.19, 1.52) | <0.001 | 1.36 (1.13, 1.62) | 0.001 |
| High genetic risk |  |  |  |  |
| 1.5 hours | 1.23 (1.05, 1.43) | 0.009 | 1.37 (1.10, 1.70) | 0.005 |
| below 1.5 hours | 1.37 (1.19, 1.57) | <0.001 | 1.35 (1.10, 1.67) | 0.004 |
| above 1.5 hours | 1.42 (1.25, 1.60) | <0.001 | 1.46 (1.22, 1.74) | <0.001 |

Abbreviation: HR, hazard ratio; 95% CI, 95% confidence interval. Adjusted for age, education, Townsend deprivation index, smoking status, drinking status, body mass index, total physical activity, sleep duration, fracture history, vitamin D supplement, hearing loss, use of sun/UV protection, PM_2.5_, hypertension, and hyperglycemia.

**Table S4. Association of genetic risk and sunlight exposure with incident depression excluding the missing information on covariates.**

| Subgroup | HR (95% CI) | *P* Value |
| --- | --- | --- |
| Summer |  |  |
| Low genetic risk |  |  |
| 2 hours | 1 (Ref.) |  |
| below 2 hours | 1.11 (0.99, 1.25) | 0.085 |
| above 2 hours | 1.21 (1.11, 1.31) | <0.001 |
| Intermediate genetic risk |  |  |
| 2 hours | 1.23 (1.11, 1.36) | <0.001 |
| below 2 hours | 1.36 (1.21, 1.52) | <0.001 |
| above 2 hours | 1.28 (1.18, 1.40) | <0.001 |
| High genetic risk |  |  |
| 2 hours | 1.23 (1.11, 1.35) | <0.001 |
| below 2 hours | 1.33 (1.19, 1.49) | <0.001 |
| above 2 hours | 1.36 (1.25, 1.48) | <0.001 |
| Winter |  |  |
| Low genetic risk |  |  |
| 1 hour | 1 (Ref.) |  |
| below 1 hour | 1.08 (0.98, 1.19) | 0.116 |
| above 1 hour | 1.14 (1.06, 1.23) | 0.001 |
| Intermediate genetic risk |  |  |
| 1 hour | 1.18 (1.09, 1.28) | <0.001 |
| below 1 hour | 1.29 (1.18, 1.41) | <0.001 |
| above 1 hour | 1.19 (1.10, 1.28) | <0.001 |
| High genetic risk |  |  |
| 1 hour | 1.17 (1.08, 1.26) | <0.001 |
| below 1 hour | 1.21 (1.10, 1.32) | <0.001 |
| above 1 hour | 1.32 (1.23, 1.42) | <0.001 |
| Average |  |  |
| Low genetic risk |  |  |
| 1.5 hours | 1 (Ref.) |  |
| below 1.5 hours | 1.14 (1.01, 1.30) | 0.038 |
| above 1.5 hours | 1.29 (1.16, 1.44) | <0.001 |
| Intermediate genetic risk |  |  |
| 1.5 hours | 1.33 (1.16, 1.52) | <0.001 |
| below 1.5 hours | 1.39 (1.23, 1.58) | <0.001 |
| above 1.5 hours | 1.37 (1.23, 1.52) | <0.001 |
| High genetic risk |  |  |
| 1.5 hours | 1.25 (1.09, 1.43) | <0.001 |
| below 1.5 hours | 1.37 (1.22, 1.55) | <0.001 |
| above 1.5 hours | 1.46 (1.31, 1.62) | <0.001 |

Abbreviation: HR, hazard ratio; 95% CI, 95% confidence interval. Adjusted for age, gender, education, Townsend deprivation index, smoking status, drinking status, body mass index, total physical activity, sleep duration, fracture history, vitamin D supplement, hearing loss, use of sun/UV protection, PM_2.5_, hypertension, and hyperglycemia.

**Table S5. Risk of incident depression according to genetic risk and outdoor light time after excluding the first 2 years of developing depression.**

| Subgroup | HR (95% CI) | *P* Value |
| --- | --- | --- |
| Summer |  |  |
| Low genetic risk |  |  |
| 2 hours | 1 (Ref.) |  |
| below 2 hours | 1.09 (0.97, 1.22) | 0.170 |
| above 2 hours | 1.17 (1.08, 1.27) | <0.001 |
| Intermediate genetic risk |  |  |
| 2 hours | 1.18 (1.07, 1.31) | 0.001 |
| below 2 hours | 1.39 (1.25, 1.55) | <0.001 |
| above 2 hours | 1.26 (1.16, 1.36) | <0.001 |
| High genetic risk |  |  |
| 2 hours | 1.22 (1.10, 1.34) | <0.001 |
| below 2 hours | 1.32 (1.18, 1.47) | <0.001 |
| above 2 hours | 1.34 (1.23, 1.45) | <0.001 |
| Winter |  |  |
| Low genetic risk |  |  |
| 1 hour | 1 (Ref.) |  |
| below 1 hour | 1.07 (0.98, 1.18) | 0.131 |
| above 1 hour | 1.14 (1.06, 1.22) | 0.001 |
| Intermediate genetic risk |  |  |
| 1 hour | 1.17 (1.08, 1.27) | <0.001 |
| below 1 hour | 1.32 (1.21, 1.44) | <0.001 |
| above 1 hour | 1.19 (1.11, 1.28) | <0.001 |
| High genetic risk |  |  |
| 1 hour | 1.17 (1.08, 1.27) | <0.001 |
| below 1 hour | 1.24 (1.14, 1.36) | <0.001 |
| above 1 hour | 1.32 (1.23, 1.42) | <0.001 |
| Average |  |  |
| Low genetic risk |  |  |
| 1.5 hours | 1 (Ref.) |  |
| below 1.5 hours | 1.10 (0.97, 1.25) | 0.122 |
| above 1.5 hours | 1.23 (1.11, 1.37) | <0.001 |
| Intermediate genetic risk |  |  |
| 1.5 hours | 1.25 (1.09, 1.42) | 0.001 |
| below 1.5 hours | 1.39 (1.23, 1.56) | <0.001 |
| above 1.5 hours | 1.31 (1.18, 1.46) | <0.001 |
| High genetic risk |  |  |
| 1.5 hours | 1.25 (1.10, 1.42) | 0.001 |
| below 1.5 hours | 1.33 (1.19, 1.50) | <0.001 |
| above 1.5 hours | 1.41 (1.27, 1.56) | <0.001 |

Abbreviation: HR, hazard ratio; 95% CI, 95% confidence interval. Adjusted for age, gender, education, Townsend deprivation index, smoking status, drinking status, body mass index, total physical activity, sleep duration, fracture history, vitamin D supplement, hearing loss, use of sun/UV protection, PM_2.5_, hypertension, and hyperglycemia.

**Table S6. Risk of incident depression according to genetic risk and outdoor light time after excluding participants whose average outdoor light time was over 2-fold of standard deviation.**

| Subgroup | HR (95% CI) | *P* Value |
| --- | --- | --- |
| Summer |  |  |
| Low genetic risk |  |  |
| 2 hours | 1 (Ref.) |  |
| below 2 hours | 1.12 (1.01, 1.26) | 0.044 |
| above 2 hours | 1.18 (1.09, 1.28) | <0.001 |
| Intermediate genetic risk |  |  |
| 2 hours | 1.21 (1.10, 1.34) | <0.001 |
| below 2 hours | 1.43 (1.28, 1.59) | <0.001 |
| above 2 hours | 1.28 (1.18, 1.38) | <0.001 |
| High genetic risk |  |  |
| 2 hours | 1.25 (1.13, 1.37) | <0.001 |
| below 2 hours | 1.34 (1.20, 1.49) | <0.001 |
| above 2 hours | 1.34 (1.24, 1.45) | <0.001 |
| Winter |  |  |
| Low genetic risk |  |  |
| 1 hour | 1 (Ref.) |  |
| below 1 hour | 1.08 (0.98, 1.18) | 0.108 |
| above 1 hour | 1.12 (1.04, 1.21) | 0.002 |
| Intermediate genetic risk |  |  |
| 1 hour | 1.18 (1.10, 1.28) | <0.001 |
| below 1 hour | 1.33 (1.22, 1.45) | <0.001 |
| above 1 hour | 1.19 (1.11, 1.28) | <0.001 |
| High genetic risk |  |  |
| 1 hour | 1.17 (1.08, 1.26) | <0.001 |
| below 1 hour | 1.24 (1.14, 1.35) | <0.001 |
| above 1 hour | 1.31 (1.22, 1.41) | <0.001 |
| Average |  |  |
| Low genetic risk |  |  |
| 1.5 hours | 1 (Ref.) |  |
| below 1.5 hours | 1.14 (1.01, 1.28) | 0.036 |
| above 1.5 hours | 1.25 (1.13, 1.39) | <0.001 |
| Intermediate genetic risk |  |  |
| 1.5 hours | 1.30 (1.14, 1.47) | <0.001 |
| below 1.5 hours | 1.43 (1.27, 1.60) | <0.001 |
| above 1.5 hours | 1.35 (1.22, 1.49) | <0.001 |
| High genetic risk |  |  |
| 1.5 hours | 1.27 (1.12, 1.44) | <0.001 |
| below 1.5 hours | 1.36 (1.22, 1.53) | <0.001 |
| above 1.5 hours | 1.43 (1.29, 1.58) | <0.001 |

Abbreviation: HR, hazard ratio; 95% CI, 95% confidence interval. Adjusted for age, gender, education, Townsend deprivation index, smoking status, drinking status, body mass index, total physical activity, sleep duration, fracture history, vitamin D supplement, hearing loss, use of sun/UV protection, PM_2.5_, hypertension, and hyperglycemia.

**Table S7. Risk of incident depression according to genetic risk and outdoor light time after further adjusting for antidepressant use.**

| Subgroup | HR (95% CI) | *P* Value |
| --- | --- | --- |
| Summer |  |  |
| Low genetic risk |  |  |
| 2 hours | 1 (Ref.) |  |
| below 2 hours | 1.12 (1.00, 1.25) | 0.058 |
| above 2 hours | 1.19 (1.10, 1.29) | <0.001 |
| Intermediate genetic risk |  |  |
| 2 hours | 1.22 (1.11, 1.35) | <0.001 |
| below 2 hours | 1.41 (1.27, 1.57) | <0.001 |
| above 2 hours | 1.27 (1.17, 1.37) | <0.001 |
| High genetic risk |  |  |
| 2 hours | 1.24 (1.13, 1.36) | <0.001 |
| below 2 hours | 1.31 (1.18, 1.45) | <0.001 |
| above 2 hours | 1.33 (1.23, 1.44) | <0.001 |
| Winter |  |  |
| Low genetic risk |  |  |
| 1 hour | 1 (Ref.) |  |
| below 1 hour | 1.05 (0.96, 1.15) | 0.313 |
| above 1 hour | 1.13 (1.06, 1.22) | 0.001 |
| Intermediate genetic risk |  |  |
| 1 hour | 1.18 (1.09, 1.27) | <0.001 |
| below 1 hour | 1.28 (1.18, 1.40) | <0.001 |
| above 1 hour | 1.18 (1.10, 1.26) | <0.001 |
| High genetic risk |  |  |
| 1 hour | 1.15 (1.07, 1.24) | <0.001 |
| below 1 hour | 1.19 (1.09, 1.29) | <0.001 |
| above 1 hour | 1.30 (1.21, 1.39) | <0.001 |
| Average |  |  |
| Low genetic risk |  |  |
| 1.5 hours | 1 (Ref.) |  |
| below 1.5 hours | 1.13 (1.00, 1.27) | 0.049 |
| above 1.5 hours | 1.27 (1.15, 1.41) | <0.001 |
| Intermediate genetic risk |  |  |
| 1.5 hours | 1.30 (1.15, 1.48) | <0.001 |
| below 1.5 hours | 1.42 (1.26, 1.59) | <0.001 |
| above 1.5 hours | 1.34 (1.21, 1.49) | <0.001 |
| High genetic risk |  |  |
| 1.5 hours | 1.27 (1.12, 1.44) | <0.001 |
| below 1.5 hours | 1.34 (1.20, 1.51) | <0.001 |
| above 1.5 hours | 1.42 (1.28, 1.57) | <0.001 |
| Abbreviation: HR, hazard ratio; 95% CI, 95% confidence interval. Adjusted for age, gender, education, Townsend deprivation index, smoking status, drinking status, body mass index, total physical activity, sleep duration, fracture history, vitamin D supplement, hearing loss, use of sun/UV protection, PM2.5, hypertension, hyperglycemia, and antidepressant use. | | |

**Table S8. Risk of incident depression according to time spent in outdoor light based on individuals with or without sun/UV protection.**

| Time spent  in outdoor light | Sun/UV protection | | | |
| --- | --- | --- | --- | --- |
|  | No or occasionally | | Yes | |
|  | HR (95% CI) | *P* Value | HR (95% CI) | *P* Value |
| Summer |  |  |  |  |
| 2 hours | 1 (Ref.) |  | 1 (Ref.) |  |
| below 2 hours | 1.20 (1.10, 1.32) | <0.001 | **1.04 (0.96, 1.13)** | **0.320** |
| above 2 hours | 1.16 (1.08, 1.24) | <0.001 | 1.07 (1.01, 1.13) | 0.025 |
| Winter |  |  |  |  |
| 1 hour | 1 (Ref.) |  | 1 (Ref.) |  |
| below 1 hour | 1.14 (1.06, 1.23) | 0.001 | **1.06 (0.99, 1.13)** | **0.080** |
| above 1 hour | 1.11 (1.05, 1.18) | 0.001 | 1.10 (1.04, 1.15) | 0.001 |
| Average |  |  |  |  |
| 1.5 hours | 1 (Ref.) |  | 1 (Ref.) |  |
| below 1.5 hours | 1.16 (1.05, 1.28) | 0.003 | **1.05 (0.97, 1.15)** | **0.239** |
| above 1.5 hours | 1.19 (1.09, 1.30) | <0.001 | 1.12 (1.04, 1.20) | 0.002 |

Abbreviation: HR, hazard ratio; 95% CI, 95% confidence interval. Adjusted for age, gender, education, Townsend deprivation index, smoking status, drinking status, body mass index, total physical activity, sleep duration, fracture history, vitamin D supplement, hearing loss, PM2.5, hypertension, hyperglycemia, and genetic risk.

**Table S9. Risk of incident depression according to time spent in outdoor light based on individuals who used or did not vitamin D supplements.**

| Time spent  in outdoor light | Without vitamin D supplements | | Vitamin D supplements | |
| --- | --- | --- | --- | --- |
|  | HR (95% CI) | *P* Value | HR (95% CI) | *P* Value |
| Summer |  |  |  |  |
| 2 hours | 1 (Ref.) |  | 1 (Ref.) |  |
| below 2 hours | 1.11 (1.04, 1.18) | 0.002 | **1.09 (0.84, 1.41)** | **0.530** |
| above 2 hours | 1.11 (1.06, 1.17) | <0.001 | **0.96 (0.78, 1.17)** | **0.673** |
| Winter |  |  |  |  |
| 1 hour | 1 (Ref.) |  | 1 (Ref.) |  |
| below 1 hour | 1.08 (1.03, 1.14) | 0.002 | **1.21 (0.97, 1.51)** | **0.098** |
| above 1 hour | 1.10 (1.05, 1.14) | <0.001 | **1.14 (0.95, 1.38)** | **0.166** |
| Average |  |  |  |  |
| 1.5 hours | 1 (Ref.) |  | 1 (Ref.) |  |
| below 1.5 hours | 1.09 (1.02, 1.16) | 0.012 | **1.27 (0.95, 1.71)** | **0.111** |
| above 1.5 hours | 1.14 (1.08, 1.21) | <0.001 | **1.22 (0.94, 1.58)** | **0.143** |

Abbreviation: HR, hazard ratio; 95% CI, 95% confidence interval. Adjusted for age, gender, education, Townsend deprivation index, smoking status, drinking status, body mass index, total physical activity, sleep duration, fracture history, sun/UV protection, hearing loss, PM2.5, hypertension, hyperglycemia, and genetic risk.

**Table S10. Risk of incident depression according to time spent in outdoor light after further adjusting for UK Biobank assessment centers.**

| Time spent  in outdoor light | Model 1 | | Model 2 | |
| --- | --- | --- | --- | --- |
|  | HR (95% CI) | *P* Value | HR (95% CI) | *P* Value |
| Summer |  |  |  |  |
| 2 hours | 1 (Ref.) |  | 1 (Ref.) |  |
| below 2 hours | 1.14 (1.08, 1.21) | <0.001 | 1.11 (1.04, 1.18) | 0.001 |
| above 2 hours | 1.19 (1.14, 1.24) | <0.001 | 1.12 (1.07, 1.17) | <0.001 |
| Winter |  |  |  |  |
| 1 hour | 1 (Ref.) |  | 1 (Ref.) |  |
| below 1 hour | 1.14 (1.08, 1.20) | <0.001 | 1.10 (1.04, 1.15) | <0.001 |
| above 1 hour | 1.19 (1.14, 1.23) | <0.001 | 1.09 (1.05, 1.14) | <0.001 |
| Average |  |  |  |  |
| 1.5 hours | 1 (Ref.) |  | 1 (Ref.) |  |
| below 1.5 hours | 1.13 (1.06, 1.21) | <0.001 | 1.08 (1.01, 1.15) | 0.020 |
| above 1.5 hours | 1.24 (1.18, 1.31) | <0.001 | 1.13 (1.08, 1.20) | <0.001 |
| Model 1 was unadjusted; Model 2 adjusted for age, gender, education, Townsend deprivation index, smoking status, drinking status, body mass index, total physical activity, sleep duration, fracture history, vitamin D supplement, hearing loss, use of sun/UV protection, PM2.5, hypertension, hyperglycemia, genetic risk, and UK Biobank assessment centers. | | | | |

**Table S11. Risk of incident depression according to genetic risk and time spent in outdoor light after further adjusting for UK Biobank assessment centers.**

| Subgroup | HR (95% CI) | *P* Value |
| --- | --- | --- |
| Summer |  |  |
| Low genetic risk |  |  |
| 2 hours | 1 (Ref.) |  |
| below 2 hours | 1.11 (0.99, 1.24) | 0.074 |
| above 2 hours | 1.22 (1.12, 1.32) | <0.001 |
| Intermediate genetic risk | |  |
| 2 hours | 1.21 (1.10, 1.34) | <0.001 |
| below 2 hours | 1.41 (1.27, 1.56) | <0.001 |
| above 2 hours | 1.29 (1.19, 1.40) | <0.001 |
| High genetic risk |  |  |
| 2 hours | 1.23 (1.12, 1.36) | <0.001 |
| below 2 hours | 1.31 (1.18, 1.46) | <0.001 |
| above 2 hours | 1.36 (1.25, 1.47) | <0.001 |
| Winter |  |  |
| Low genetic risk |  |  |
| 1 hour | 1 (Ref.) |  |
| below 1 hour | 1.09 (0.99, 1.19) | 0.071 |
| above 1 hour | 1.14 (1.06, 1.23) | <0.001 |
| Intermediate genetic risk | |  |
| 1 hour | 1.18 (1.09, 1.27) | <0.001 |
| below 1 hour | 1.33 (1.22, 1.45) | <0.001 |
| above 1 hour | 1.19 (1.11, 1.27) | <0.001 |
| High genetic risk |  |  |
| 1 hour | 1.15 (1.07, 1.25) | <0.001 |
| below 1 hour | 1.23 (1.13, 1.34) | <0.001 |
| above 1 hour | 1.31 (1.22, 1.40) | <0.001 |
| Average |  |  |
| Low genetic risk |  |  |
| 1.5 hours | 1 (Ref.) |  |
| below 1.5 hours | 1.12 (0.99, 1.26) | 0.077 |
| above 1.5 hours | 1.27 (1.15, 1.41) | <0.001 |
| Intermediate genetic risk | |  |
| 1.5 hours | 1.29 (1.14, 1.47) | <0.001 |
| below 1.5 hours | 1.40 (1.24, 1.57) | <0.001 |
| above 1.5 hours | 1.35 (1.22, 1.49) | <0.001 |
| High genetic risk |  |  |
| 1.5 hours | 1.26 (1.11, 1.43) | <0.001 |
| below 1.5 hours | 1.32 (1.18, 1.48) | <0.001 |
| above 1.5 hours | 1.43 (1.29, 1.58) | <0.001 |
| Adjusted for age, gender, education, Townsend deprivation index, smoking status, drinking status, body mass index, total physical activity, sleep duration, fracture history, vitamin D supplement, hearing loss, use of sun/UV protection, PM2.5, hypertension, hyperglycemia, and UK Biobank assessment centers. | | |

**Table S12. Risk of incident depression according to genetic risk and time spent in outdoor light after further adjusting for employment.**

| Subgroup | HR (95% CI) | *P* Value |
| --- | --- | --- |
| Summer |  |  |
| Low genetic risk |  |  |
| 2 hours | 1 (Ref.) |  |
| below 2 hours | 1.13 (1.01, 1.26) | 0.038 |
| above 2 hours | 1.14 (1.05, 1.24) | 0.001 |
| Intermediate genetic risk |  |  |
| 2 hours | 1.22 (1.11, 1.35) | <0.001 |
| below 2 hours | 1.43 (1.29, 1.59) | <0.001 |
| above 2 hours | 1.22 (1.13, 1.32) | <0.001 |
| High genetic risk |  |  |
| 2 hours | 1.24 (1.13, 1.37) | <0.001 |
| below 2 hours | 1.33 (1.20, 1.48) | <0.001 |
| above 2 hours | 1.29 (1.19, 1.39) | <0.001 |
| Winter |  |  |
| Low genetic risk |  |  |
| 1 hour | 1 (Ref.) |  |
| below 1 hour | 1.07 (0.97, 1.17) | 0.159 |
| above 1 hour | 1.11 (1.03, 1.19) | 0.004 |
| Intermediate genetic risk |  |  |
| 1 hour | 1.18 (1.10, 1.28) | <0.001 |
| below 1 hour | 1.31 (1.20, 1.43) | <0.001 |
| above 1 hour | 1.16 (1.08, 1.24) | <0.001 |
| High genetic risk |  |  |
| 1 hour | 1.17 (1.08, 1.26) | <0.001 |
| below 1 hour | 1.22 (1.12, 1.32) | <0.001 |
| above 1 hour | 1.28 (1.20, 1.37) | <0.001 |
| Average |  |  |
| Low genetic risk |  |  |
| 1.5 hours | 1 (Ref.) |  |
| below 1.5 hours | 1.13 (1.01, 1.28) | 0.041 |
| above 1.5 hours | 1.22 (1.10, 1.35) | <0.001 |
| Intermediate genetic risk |  |  |
| 1.5 hours | 1.30 (1.14, 1.47) | <0.001 |
| below 1.5 hours | 1.42 (1.27, 1.60) | <0.001 |
| above 1.5 hours | 1.30 (1.17, 1.43) | <0.001 |
| High genetic risk |  |  |
| 1.5 hours | 1.27 (1.12, 1.44) | <0.001 |
| below 1.5 hours | 1.35 (1.20, 1.52) | <0.001 |
| above 1.5 hours | 1.38 (1.25, 1.52) | <0.001 |
| Abbreviation: HR, hazard ratio; 95% CI, 95% confidence interval. Adjusted for age, gender, education, Townsend deprivation index, smoking status, drinking status, body mass index, total physical activity, sleep duration, fracture history, vitamin D supplement, hearing loss, use of sun/UV protection, PM2.5, hypertension, hyperglycemia, and employment. | | |

**Table S13. Risk of incident depression according to genetic risk and time spent in outdoor light after further adjusting for living environment (urban and rural areas).**

| Subgroup | HR (95% CI) | *P* Value |
| --- | --- | --- |
| Summer |  |  |
| Low genetic risk |  |  |
| 2 hours | 1 (Ref.) |  |
| below 2 hours | 1.12 (1.00, 1.25) | 0.057 |
| above 2 hours | 1.19 (1.10, 1.30) | <0.001 |
| Intermediate genetic risk |  |  |
| 2 hours | 1.21 (1.10, 1.34) | <0.001 |
| below 2 hours | 1.42 (1.28, 1.58) | <0.001 |
| above 2 hours | 1.28 (1.18, 1.39) | <0.001 |
| High genetic risk |  |  |
| 2 hours | 1.24 (1.13, 1.37) | <0.001 |
| below 2 hours | 1.33 (1.20, 1.48) | <0.001 |
| above 2 hours | 1.35 (1.24, 1.46) | <0.001 |
| Winter |  |  |
| Low genetic risk |  |  |
| 1 hour | 1 (Ref.) |  |
| below 1 hour | 1.08 (0.98, 1.18) | 0.107 |
| above 1 hour | 1.13 (1.06, 1.22) | 0.001 |
| Intermediate genetic risk |  |  |
| 1 hour | 1.18 (1.10, 1.28) | <0.001 |
| below 1 hour | 1.33 (1.22, 1.44) | <0.001 |
| above 1 hour | 1.19 (1.11, 1.27) | <0.001 |
| High genetic risk |  |  |
| 1 hour | 1.16 (1.08, 1.26) | <0.001 |
| below 1 hour | 1.24 (1.13, 1.35) | <0.001 |
| above 1 hour | 1.31 (1.22, 1.41) | <0.001 |
| Average |  |  |
| Low genetic risk |  |  |
| 1.5 hours | 1 (Ref.) |  |
| below 1.5 hours | 1.12 (0.99, 1.26) | 0.066 |
| above 1.5 hours | 1.26 (1.14, 1.40) | <0.001 |
| Intermediate genetic risk |  |  |
| 1.5 hours | 1.30 (1.14, 1.47) | <0.001 |
| below 1.5 hours | 1.41 (1.25, 1.58) | <0.001 |
| above 1.5 hours | 1.34 (1.21, 1.49) | <0.001 |
| High genetic risk |  |  |
| 1.5 hours | 1.27 (1.12, 1.44) | <0.001 |
| below 1.5 hours | 1.34 (1.19, 1.50) | <0.001 |
| above 1.5 hours | 1.43 (1.29, 1.58) | <0.001 |
| Abbreviation: HR, hazard ratio; 95% CI, 95% confidence interval. Adjusted for age, gender, education, Townsend deprivation index, smoking status, drinking status, body mass index, total physical activity, sleep duration, fracture history, vitamin D supplement, hearing loss, use of sun/UV protection, PM2.5, hypertension, hyperglycemia, and living environment. | | |

| **Table S14. Risk of incident depression according to time spent in outdoor light after including extreme hours spent in outdoor light.** | | | | |
| --- | --- | --- | --- | --- |
| Time spent  in outdoor light | Model 1 | | Model 2 | |
|  | HR (95% CI) | *P* Value | HR (95% CI) | *P* Value |
| Summer |  |  |  |  |
| 2 hours | 1 (Ref.) |  | 1 (Ref.) |  |
| < 2 hours | 1.14 (1.08, 1.21) | <0.001 | 1.12 (1.05, 1.19) | <0.001 |
| > 2 and ≤ 16 hours | 1.19 (1.14, 1.24) | <0.001 | 1.10 (1.06, 1.15) | <0.001 |
| > 16 hours | 2.32 (1.34, 4.00) | 0.003 | 1.83 (1.06, 3.15) | 0.030 |
| Winter |  |  |  |  |
| 1 hour | 1 (Ref.) |  | 1 (Ref.) |  |
| < 1 hour | 1.14 (1.09, 1.20) | <0.001 | 1.07 (1.02, 1.13) | 0.004 |
| > 1 and ≤ 8 hours | 1.19 (1.14, 1.23) | <0.001 | 1.09 (1.04, 1.13) | <0.001 |
| > 8 hours | 1.30 (1.12, 1.51) | <0.001 | 1.06 (0.91, 1.23) | 0.478 |
| Average |  |  |  |  |
| 1.5 hours | 1 (Ref.) |  | 1 (Ref.) |  |
| < 1.5 hours | 1.13 (1.06, 1.21) | <0.001 | 1.08 (1.02, 1.16) | 0.013 |
| > 1.5 and ≤ 12 hours | 1.25 (1.18, 1.32) | <0.001 | 1.13 (1.07, 1.19) | <0.001 |
| > 12 hours | 1.66 (1.14, 2.41) | 0.008 | 1.28 (0.88, 1.87) | 0.195 |
| Abbreviation: HR, hazard ratio; 95% CI, 95% confidence interval. Model 1 was unadjusted; Model 2 adjusted for age, gender, education, Townsend deprivation index, smoking status, drinking status, body mass index, total physical activity, sleep duration, fracture history, vitamin D supplement, hearing loss, use of sun/UV protection, PM2.5, hypertension, hyperglycemia, and genetic risk. | | | | |

**Table S15. Association between covariates and the risk of depression.**

| Covariates | HR (95% CI) | *P* value |
| --- | --- | --- |
| Age | 1.00 (1.00, 1.00) | 0.677 |
| Gender |  |  |
| Female | 1.00 (Ref.) |  |
| Male | 0.62 (0.60, 0.65) | <0.001 |
| Townsend deprivation index, mean (SD) | 1.05 (1.05, 1.06) | <0.001 |
| Education |  |  |
| College or University degree | 1.00 (Ref.) |  |
| Upper secondary | 1.19 (1.12, 1.27) | <0.001 |
| Lower secondary | 1.36 (1.29, 1.42) | <0.001 |
| Vocational | 1.48 (1.38, 1.59) | <0.001 |
| Other | 1.56 (1.48, 1.64) | <0.001 |
| Smoking status |  |  |
| Never | 1.00 (Ref.) |  |
| Previous | 1.23 (1.18, 1.28) | <0.001 |
| Current | 1.85 (1.76, 1.94) | <0.001 |
| Drinking status |  |  |
| Never | 1.00 (Ref.) |  |
| Previous | 1.78 (1.66, 1.91) | <0.001 |
| Current | 1.17 (1.08, 1.27) | <0.001 |
| Body mass index (kg/m^2^), mean (SD) | 1.32 (1.27, 1.37) | <0.001 |
| Total physical activity, MET-min/week, mean (SD) | 1.00 (1.00, 1.00) | 0.045 |
| Sleep duration, hours/day, mean (SD) | 0.98 (0.96, 0.99) | 0.003 |
| PM_2.5_, ug/m^3^, mean (SD) | 1.02 (1.00, 1.04) | 0.023 |
| Vitamin D supplement |  |  |
| No | 1.00 (Ref.) |  |
| Yes | 1.16 (1.07, 1.26) | <0.001 |
| Use of sun/UV protection |  |  |
| No or occasionally | 1.00 (Ref.) |  |
| Yes | 0.80 (0.76, 0.85) | <0.001 |
| Do not go out in the sunshine | 1.29 (1.08, 1.54) | 0.005 |
| Fracture history |  |  |
| No | 1.00 (Ref.) |  |
| Yes | 1.32 (1.25, 1.38) | <0.001 |
| Hearing loss |  |  |
| No | 1.00 (Ref.) |  |
| Yes | 1.43 (1.38, 1.48) | <0.001 |
| Hypertension |  |  |
| No | 1.00 (Ref.) |  |
| Yes | 0.98 (0.94, 1.01) | 0.212 |
| Hyperglycemia |  |  |
| No | 1.00 (Ref.) |  |
| Yes | 1.32 (1.25, 1.39) | <0.001 |

**Figure S1. Flowchart for the selection of the analyzed study population from UK Biobank.**

- 1,347 with losing follow–up information.

453,255 White Participants

- 12,881 with the missing data on genetic risk.
- 30,232 participants who reported “do not know” or “prefer not to answer” of the information on outdoor light.
- 4,647 with the extreme values on outdoor light.
- 24,519 with the history of depression

502,507 Baseline participants with available data

380,976 Participants were included

- 13,636 depression cases
- 367,340 depression–free participants

.

**Figure S2. Percentage of genetic risk according to outdoor light categories.**


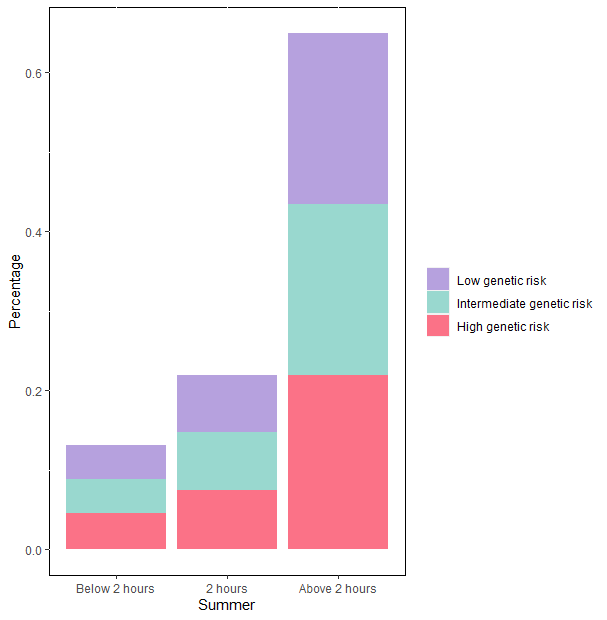

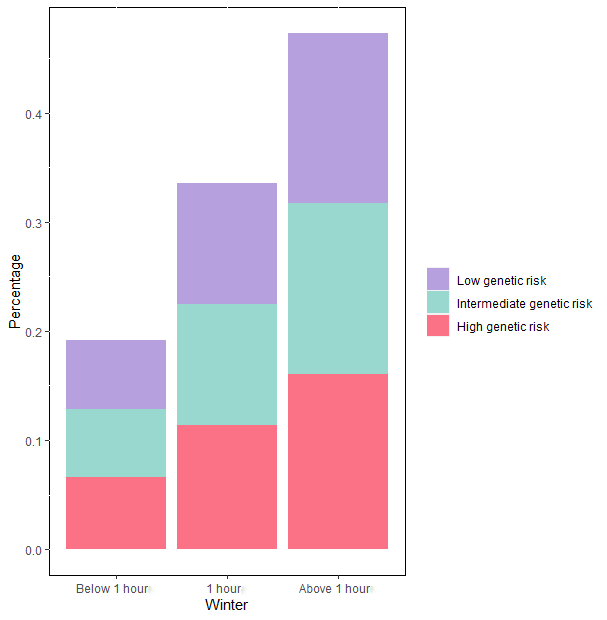

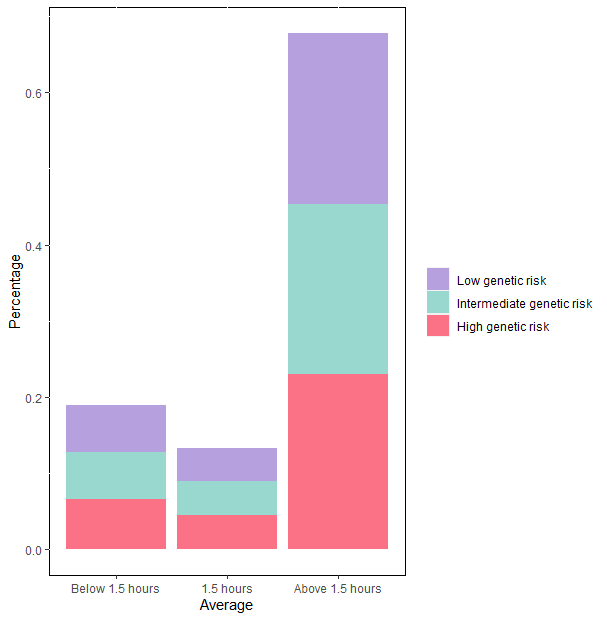

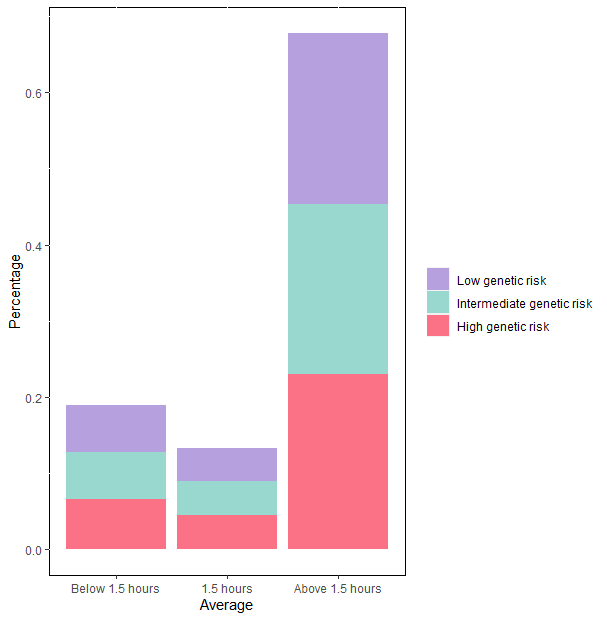


**Figure S3. The correlation between gene risk and incident depression during follow-up.**


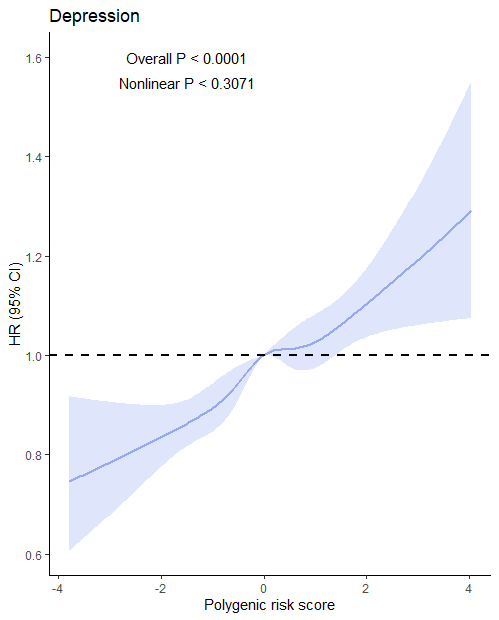


Abbreviation: HR, hazard ratio; 95% CI, 95% confidence interval.

Adjusted for age, gender, education, Townsend deprivation index, smoking status, drinking status, body mass index, total physical activity, sleep duration, fracture history, vitamin D supplement, hearing loss, use of sun/UV protection, PM_2.5_, hypertension, and hyperglycemia.

**Figure S4. The dose-response association between time spent in outdoor light in winter and depression risk after dividing individuals spent less 1 hour into a group.**

**
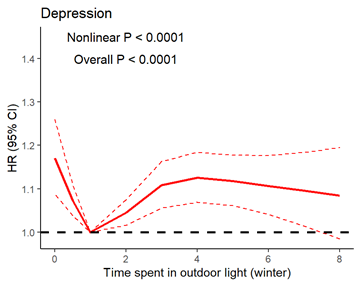
**

**Figure S5. Dose-response association between time spent in outdoor light and the risk of depression after including extreme hours spent in outdoor light.**


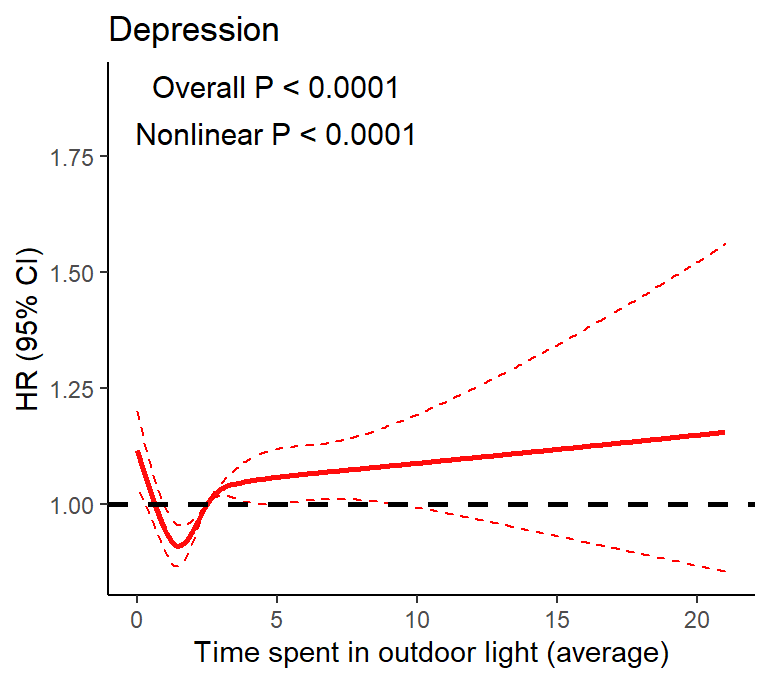

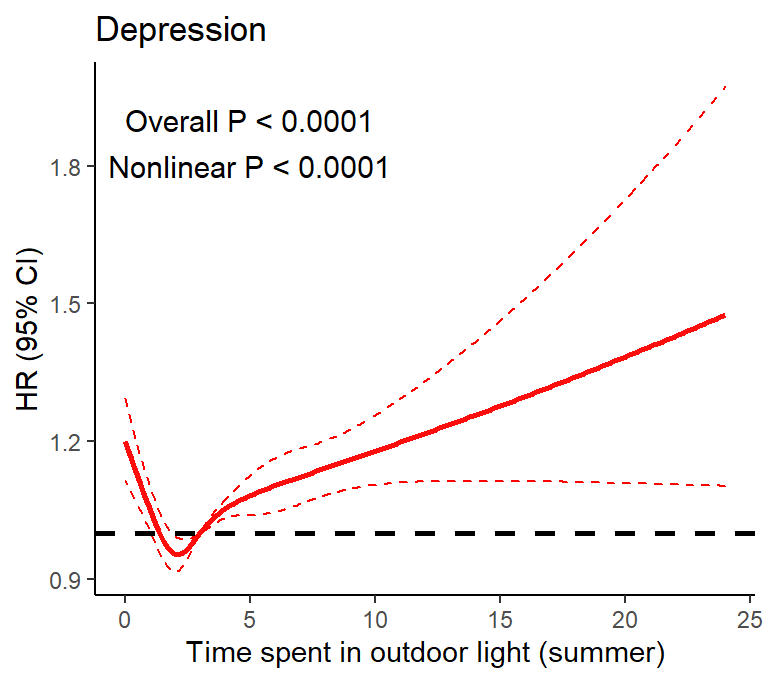

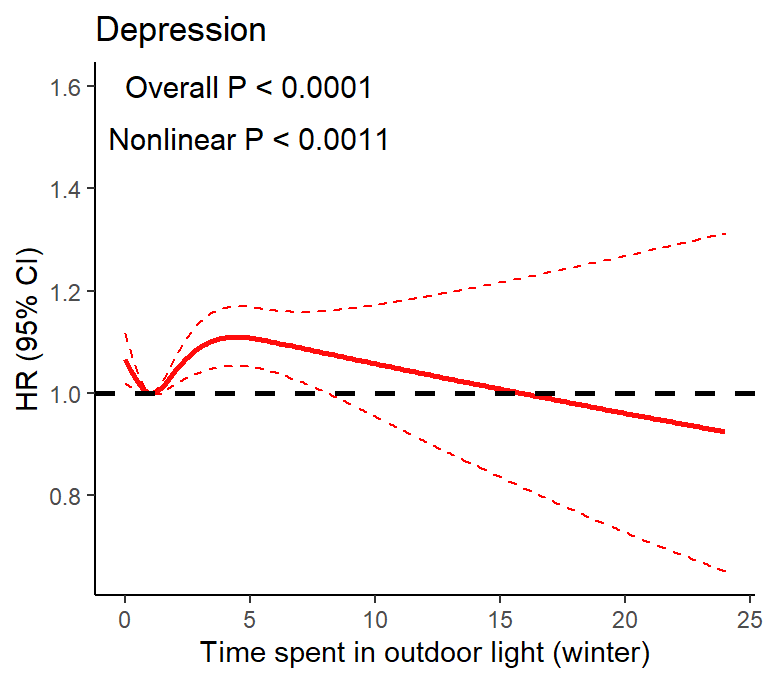


**References:**

1. Ma LZ, Ma YH, Ou YN, Chen SD, Yang L, Dong Q *et al.*. Time spent in outdoor light is associated with the risk of dementia: a prospective cohort study of 362094 participants. *BMC MED* 2022 2022-4-25; **20**(1)**:** 132.
